# The sieve-element endoplasmic reticulum: A focal point of phytoplasma-host plant interaction?

**DOI:** 10.3389/fmicb.2023.1030414

**Published:** 2023-02-02

**Authors:** Rita Musetti, Laura Pagliari, Giovanni Mian, Fernando R. De Oliveira Cantao, Chiara Bernardini, Simonetta Santi, Aart J. E. van Bel

**Affiliations:** ^1^Department of Land, Environment, Agriculture and Forestry (TESAF), Università di Padova, via dell' Università, Legnaro, Italy; ^2^Department of Agricultural, Food, Environmental and Animal Sciences, University of Udine, via delle Scienze, Udine, Italy; ^3^Institute of Phytopathology, Justus-Liebig University, Giessen, Germany

**Keywords:** Arabidopsis, endoplasmic reticulum, phytoplasma, phytoplasma-host interaction, sieve element, pore-plasmodesma units, sieve-element ER docking sites, unfolded protein response

## Abstract

The rough endoplasmic reticulum (r-ER) is of paramount importance for adaptive responses to biotic stresses due to an increased demand for *de novo* synthesis of immunity-related proteins and signaling components. In nucleate cells, disturbance of r-ER integrity and functionality leads to the “unfolded protein response” (UPR), which is an important component of innate plant immune signalling. In contrast to an abundance of reports on r-ER responses to biotic challenges, sieve-element endoplasmic reticulum (SE-ER) responses to phytoplasma infection have not been investigated. We found that morphological SE-ER changes, associated with phytoplasma infection, are accompanied by differential expression of genes encoding proteins involved in shaping and anchoring the reticulum. Phytoplasma infection also triggers an increased release of bZIP signals from the (SE-ER)/r-ER and consequent differential expression of UPR-related genes. The modified expression patterns seem to reflect a trade-off between survival of host cells, needed for the phytoplasmic biotrophic lifestyle, and phytoplasmas. Specialized plasmodesmata between sieve element and companion cell may provide a corridor for transfer of phytoplasma effectors inducing UPR-related gene expression in companion cells.

## 1. Introduction

Phytoplasmas are phytopathogenic mollicutes associated with numerous economically relevant plant diseases, worldwide (Namba, 2019). Biology of phytoplasmas and host responses to infection are still largely unknown due to the biotrophic lifestyle of these microorganisms which impedes *in vitro* studies (Jiang et al., 2019; Mapuranga et al., 2022). In plants, phytoplasmas reside exclusively in sieve elements (SEs; van Bel and Musetti, 2019; Lewis et al., 2022), highly specialized transport cells, that provide an exceptional physical and chemical environment, favored by phytoplasmas (van Bel et al., 2022). The interaction between phytoplasmas and SE components has barely been investigated thus far (van Bel and Musetti, 2019). Phytoplasmas have a low-size genome, and their survival most likely relies on plant resources, given the absence of many key genes, essential for cell metabolism (Kube et al., 2012; Oshima et al., 2013).

Mature SEs possess a plasma membrane enclosing a thin parietal cytoplasmic layer that is in open contact with the wide sieve-element lumen, as result of the absence of a tonoplast. The enucleate cytoplasmic layer contains a reduced set of organelles such as SE plastids, inactive mitochondria and conspicuous aggregates of smooth endoplasmic reticulum, named sieve-element endoplasmic reticulum (SE-ER; e.g. Ehlers et al., 2000; van Bel and Musetti, 2019). The latter exhibits ultrastructural modifications following phytoplasma infection (Buxa et al., 2015; Pagliari et al., 2016). In addition, there are indications for the occurrence of junctions between phytoplasmas and SE-ER (Pagliari et al., 2016, 2017), as reported for other mollicutes, i.e., spiroplasmas (see van Bel and Musetti, 2019). The SE-ER is part of the SE endomembrane system (Liu et al., 2022), with a unique morphology and function in SEs (Sjolund and Shih, 1983). It is linked by minute anchors, to mitochondria, SE plastids, and the plasma membrane (Ehlers et al., 2000), to prevent dragging by mass flow in the sieve tubes. These anchors might also serve to keep the SE organelles closely together, in order to facilitate exchange of compounds *via* the unstirred layer surrounding the diverse adjacent membranes.

The rough endoplasmic reticulum (r-ER) in generic, nucleate cells is composed of two morphologically distinct domains, i.e., sheets (cisternae) and tubules, which are connected by three-way junctions to create a loose polygonal structure (Kriechbaumer and Brandizzi, 2020). The SE-ER in mature SEs is mainly formed instead by stacks of membranes, appressed to the SE plasma membrane (Ehlers et al., 2000). The r-ER is responsible for synthesis, processing and sorting of proteins and harbors membrane-bound receptors and hormonal transporters, associated with plant immune responses (Jing and Wang, 2020) and the ion channeling involved in the regulation of cytosolic Ca^2+^ levels (Almeida, 2021). By contrast, knowledge on SE-ER cisternae is scant. They are believed to act as intracellular Ca^2+^-sequestration compartments (Sjolund and Shih, 1983; van Bel et al., 2014 and literature within). Moreover, SE-ER displayed acid phosphatase activity suggesting a role in cytoplasmic autolysis during SE maturation (Oparka et al., 1981). Despite the distinctions between SE-ER in SEs and r-ER in companion cells (CCs), r-ER-specific fluorochromes (Martens et al., 2006) and experiments with transformed plants carrying a His-Asp-Glu-Leu (HDEL) r-ER-retention signal (Liu et al., 2022), demonstrate physical, *via* unilaterally branched pore-plasmodesma units (PPUs), and functional SE-ER/r-ER connections, consistent with a role in protein and signal exchange between CC and SE.

The r-ER appears to act as a central regulator of immune responses in plants and animals. Disturbance of r-ER integrity and functionality following abiotic stress and pathogen attacks, leads to the so-called “unfolded protein response” (UPR), which is a crucial part of the r-ER-mediated innate plant immune signalling (Park and Park, 2019; Howell, 2021). The UPR is initiated by activation of r-ER membrane-associated molecular sensors, followed by migration of their active components to the nucleus, where they act as transcription factors (TFs) to trigger UPR gene expression (Supplementary Figure 1; Park and Park, 2019; Howell, 2021). Although UPR protein synthesis aims at restoring proteostasis within the secretory pathway, UPR signaling may cause cell death during prolonged severe stress conditions or insufficient adaptive responses (Park and Park, 2019; Howell, 2021). In contrast to the abundance of reports on UPR, triggered in the r-ER by stress factors or pathogen attacks, in different plant species (Strasser, 2018; Verchot and Pajerowska-Mukhtar, 2021), nothing is known about a possible SE-ER -mediated response to pathogenic challenges. Recently, Kloth et al. (2021) reported that SLI1, an R-protein of Arabidopsis which confers a broad-spectrum resistance to phloem-feeding insects, co-localized with the SE-ER in the parietal layer of SEs, arousing interest in the possible role(s) of the SE-ER in plant immunity.

Aim of this study was to collect data on a few key aspects of the interaction between r-ER, SE-ER and phytoplasmas. We investigated ultrastructural alterations of SE-ER in SEs and r-ERs in CCs, phloem parenchyma cells and cortical parenchyma cells and putative junctions between phytoplasmas and SER (van Bel and Musetti, 2019) in the *Arabidopsis thaliana* / ‘*Candidatus* Phytoplasma asteris’ pathosystem (Pagliari et al., 2016, 2017). Furthermore, we examined phytoplasma effects on the expression of genes encoding proteins involved in SE-ER and r-ER re-organization, such as members of class XI myosin motor proteins, which control movement and remodelling of r-ER (Griffing et al., 2014). In addition, proteins involved in endoplasmic reticulum-plasma membrane (ER-PM) anchoring and membrane lipid transfer (i.e., SYT1, VAP27-1, and NET3C, Wang et al., 2014; Siao et al., 2016) were investigated. Because processing of proteins is enhanced under stress conditions to an extent that exceeds the r-ER folding capacities (Bao et al., 2019; Park and Park, 2019), expression levels of genes encoding proteins involved in the UPR pathway in generic nucleate cells (i. e., IRE1, bZIP60, bZIP17/28, S1P, S2P, BiPs, PDIs, CNXs, and CRTs; Adams et al., 2019; Howell, 2021) were evaluated in healthy and infected Arabidopsis midrib tissues. All genes under analysis here are summarized in Table 1.

**Table 1:** List of Arabidopsis genes examined by real-time RT-PCR in this study.

**Gene**	**Locus_tag**	**NCBI description**
**UBC9**	AT4G27960	Ubiquitin conjugating enzyme 9
**TIP41**	AT4G34270	TIP41-like family protein
**SAND**	AT2G28390	SAND family protein
**UBQ10**	AT4G05320	Polyubiquitin 10
**MXI-K**	AT5G20490	Myosin family protein with Dil (alias: XIK)
**MXI-1**	AT1G17580	Myosin 1 (alias: MYA1)
**MXI-2**	AT5G43900	Myosin 2 (alias: MYA2)
**bZIP17**	AT2G40950	Basic-leucine zipper (bZIP) transcription factor family protein
**bZIP28**	AT3G10800	Basic-leucine zipper (bZIP) transcription factor family protein
**bZIP60u**	AT1G42990	Basic region/leucine zipper motif 60
**bZIP60s**		
**IRE1A**	AT2G17520	Endoribonuclease/protein kinase IRE1-like protein
**IRE1B**	AT5G24360	Inositol requiring 1–1 (alias: IRE1-1)
**S1P**	AT5G19660	SITE-1 protease
**S2P**	AT4G20310	Peptidase M50 family protein
**BIP1**	AT5G28540	Heat shock protein 70 (Hsp 70) family protein
**BIP2**	AT5G42020	Heat shock protein 70 (Hsp 70) family protein
**BIP3**	AT1G09080	Heat shock protein 70 (Hsp 70) family protein
**PDI1**	AT3G54960	PDI-like 1–3 (alias PDIL1-3, PROTEIN DISULFIDE ISOMERASE 1)
**PDI5**	AT1G21750	PDI-like 1–1 (alias PDIL1-1, PROTEIN DISULFIDE ISOMERASE 5)
**CNX1**	AT5G61790	Calnexin 1
**CNX2**	AT2G31955	Cofactor of nitrate reductase and xanthine dehydrogenase 2
**CRT1**	AT1G56340	Calreticulin 1a (alias CRT1a)
**CRT2**	AT1G09210	Calreticulin 1b (alias CRT1b)
**CRT3**	AT1G08450	Calreticulin 3
**SYT1**	AT2G20990	Synaptotagmin 1 (alias SYTA)
**VAP27-1**	AT3G60600	Vesicle associated protein (alias: VAMP/SYNAPTOBREVIN-ASSOCIATED PROTEIN 27–1)
**NET3C**	AT2G47920	Kinase interacting (KIP1-like) family protein

The results indicate a high responsiveness of SE-ER and r-ER to phytoplasma infection, expressed by a rapid remodelling of SE-ER in the enucleate SEs, and by the activation of UPR most likely in the nucleate CCs. The SE-ER/r-ER -related host immune response is seemingly modulated in a finely balanced and selective way by the biotrophic phytoplasmas, allowing the survival and viability of the SE/CC complexes despite all pathogenic effects.

## 2. Materials and methods

### 2.1. Plant material

*Arabidopsis thaliana* ecotype Columbia (Col) plants were grown at 22/20°C, under short-day conditions (9 h light/15 h dark period). As described by Pagliari et al. (2017), the fourth and fifth instars of the insect vector *Euscelidius variegatus* (Bosco et al., 1997) were infected with Chrysanthemum yellows (CY) phytoplasma (Lee et al., 2004), a strain related to ‘*Candidatus* Phytoplasma asteris’ (‘*Ca.* P. asteris’, 16SrI-B subgroup). Arabidopsis plants were exposed to three healthy (for use on control plants) or CY-infected insects for a 7d inoculation-feeding period, as reported previously (Pagliari et al., 2016). Symptoms related to CY-phytoplasma infection showed up 20 days after the end of the inoculation period (Pagliari et al., 2016). Only the symptomatic plants (treated with the CY-infectious insect vectors) tested positive for the phytoplasma presence by PCR, using the primer pair R16F2n/R16R2 (Pagliari et al., 2017), whereas the asymptomatic, control plants tested negative (not shown).

For each analysis (ultrastructure observation, phytoplasma detection and gene expression analysis), at least five infected and five healthy control *A. thaliana* plants, unless specified otherwise, were used and considered as independent biological replicates (Pagliari et al., 2017).

### 2.2. Transmission electron microscopy

From the above-mentioned plants, a 6–7 mm long midrib portion was excised from three fully expanded rosette leaves. The midrib segments were prepared for conventional transmission electron microscopy (TEM) analyses as in Buxa et al. (2015). Ultrathin sections (60–70 nm) were cut using an ultramicrotome (Reichert Leica Ultracut E ultramicrotome, Leica Microsystems, Wetzlar, Germany) and collected on 200 mesh uncoated copper grids. Sections were then stained with UAR-EMS (uranyl acetate replacement stain; Electron Microscopy Sciences, Fort Washington, PA, United States) and observed under a PHILIPS CM 10 TEM (FEI, Eindhoven, The Netherlands), operated at 80 kV, and equipped with a Megaview G3 CCD camera (EMSIS GmbH, Münster, Germany).

### 2.3. Gene expression analysis

Transcriptional regulation of Arabidopisis genes contributing to SE-ER/r-ER dynamics and spatial configurations, was analyzed in midribs of healthy and phytoplasma-infected plants using a real-time RT-PCR approach. In particular we quantified the expression level of genes encoding the class XI myosin proteins, MXI-K, MXI-1, and MXI-2, and genes encoding proteins localized on the ER-PM contact sites in plants, i.e., synaptotagmin 1 (SYT1, Schapire et al., 2008), vesicle-associated protein/synaptobrevin-associated protein 27–1 (VAP27-1) and the actin-binding protein NETWORKED 3C (NET3C) (Ueda et al., 2010, 2015; Wang et al., 2014; Pérez-Sancho et al., 2015; Siao et al., 2016; Table 1). Furthermore, the expression levels of UPR-related Arabidopsis genes (Park and Park, 2019) were investigated, i.e., those involved in the two main UPR pathways in plant, activating (i) the inositol-requiring protein 1 (IRE1)-mediated splicing of *bZIP60* mRNA (Ruberti et al., 2015) and (ii) in the proteolytic processing of bZIP17/28 (Iwata and Koizumi, 2012) by the SITE 1 PROTEASE and SITE 2 PROTEASE (S1P and S2P, Liu et al., 2007; Supplementary Figure 1; Park and Park, 2019). In case of stress, bZIP17, bZIP28, and bZIP60 transcription factors induce the expression of several protein chaperones, reducing the number of misfolded proteins (Park and Park, 2019).

One of the major players in these processes is the luminal binding protein (BiP), an ER-resident member of the stress-related heat shock protein (HSP70) family, encoded by *BiP1*, *BiP2* and *BiP3* in Arabidopsis (Koizumi, 1996). Protein disulfide isomerase (PDI), encoded by 12 genes in Arabidopsis (Thomas and van Der Hoorn, 2018), is also involved in UPR, facilitating the correct formation of disulfide bonds between cysteine residues in proteins (Zhang et al., 2018). Also engaged in the UPR are calnexins (CNX) and calreticulins (CRT), encoded in Arabidopsis, respectively, by *CNX1*, *CNX2*, and *CRT1, CRT2, CRT3* (Nelson et al., 1997). These are calcium-binding, lectin-like chaperones that recognize the terminal structure of N-linked glycans attached to proteins and retain unfolded proteins in the r-ER (Strasser, 2018).

Total RNA was extracted from about 1 g of leaf midribs using a Spectrum RNA Kit (Sigma Aldrich, St. Louis, MO, United States) following the manufacturer’s instructions. RNAs were DNase-treated and reverse-transcribed into cDNA with a QuantiTectReverse Transcription Kit (Qiagen GmbH) following the manufacturer’s instructions. Gene expression was analyzed by real-time RT-PCR on a CFX96 instrument (Bio-Rad Laboratories, Hercules, CA, USA) using the SsoFast EvaGreen Supermix (Bio-Rad Laboratories Inc., Hercules, CA, United States) and the amount of cDNA obtained from 5 ng of RNA, in a total volume of 10 μl. Primers used for real-time RT-PCR are listed in Table 2. Arabidopsis ubiquitin conjugating enzyme 9 (UBC9) was used as reference gene, being the most stably expressed among a set of four potential housekeeping genes (Tables 1, 2; Pagliari et al., 2017). Primer pair efficiency was evaluated as described by Pfaffl (2001) on the standard curves of different dilutions of pooled cDNA. Mean normalized expression (MNE) for each gene of interest (Muller et al., 2002) was calculated by normalizing its expression to the level of the *UBC9* gene. At least five individuals (plants) with three technical repeats each were used for MNE determination of each gene, in both control and infected plants. Statistical analyses of gene expression levels were performed with the Prism 7.02 software package (GraphPad Software) using an unpaired t-test.

**Table 2:** List of primers and accession number of Arabidopsis sequences used in real-time RT-PCRs in the present study.

**Gene**	**Forward primer 5’–3’**	**Reverse primer 5’–3’**	**nM**	**NCBI accession no.**
**UBC9** ^a^	TCACAATTTCCAAGGTGCTGC^b^	CGAGCAGTGGACTCGTACTT^b^	300	NM_179131.3
				NM_118934.3
**TIP41**	CCTCTTGCGATTTTGGCTGAG^b^	ACGAAGAACAGTTGGTGCCT^b^	300	NM_119592.5
**SAND**	AGATCAATCGCGGAAGGTGG^b^	TATGTCGGGACCAGGTGAGT^b^	300	NM_128399.4
**UBQ10** ^a^	CGTCTTCGTGGTGGTTTCTAA^b^	ACAAGGCCCCAAAACACAAAC^b^	300	NM_178968.5
				NM_001084884.5
				NM_001340546.1
				NM_116771.5
				NM_202787.4
				NM_001340547.1
				NM_178969.6
				NM_178970.5
**MXI-K** ^a^	ACAGCCATTGAAGTGCCAGA	TTCGCCTCTGCGGTGTTAAA	300	NM_001161252.2
				NM_001343670.1
				NM_001343671.1
**MXI-1**	AGACGTGAATGCTGCTCGTT	AGCCGAACCAACAAACTCCT	300	NM_101620.3
**MXI-2** ^a^	ACTCCAAGCAGCCAAGAACA	TCCAGGTCAGTCCTTATCCGT	300	NM_001203536.1
				NM_123757.5
				NM_001203536.1
**bZIP17**	GCTCTATCCTCTGGCTCTGC	ATGGGACCTGCAACACCTTC	300	NM_129659.3
**bZIP28**	TTCCCGGATCTTTGTGGTGG	TCAGGTGGCTACGAGATGGA	300	NM_111917.5
**bZIP60u**	AGGAGTCTGCTGTGCTCTTG	TCTGGACGTAGGAGGCAACA	300	NM_103458.3
**bZIP60s**	GAGTCTGCTGTTGGGTTCCC	TCTGGACGTAGGAGGCAACA	300	NM_103458.3
**IRE1A**	AAAGTTTTCGTCGAGGGGCA	TCCTTCGCGGATTTACGGTT	300	NM_127306.4
**IRE1B** ^a^	ATTTGAGACCGAGAGCACAAG	TATCGCTTGCATCCCGAAGA	300	NM_001203453.2
				NM_122344.5
				NM_001203454.1
**S1P** ^a^	AGGCATCAAAGGAAGACCCTG	CAGGAGCCAGTAGCAGTTGG	300	NM_121971.3
				NM_001343619.1
				NM_001343620.1
**S2P** ^a^	TGTGGTGATGGATGGGTGAC	AGTCACCCTGTGGACATACG	300	NM_001341415.1
				NM_001341416.1
				NM_001341418.1
				NM_001341412.1
				NM_001341413.1
				NM_001160784.2
				NM_001341417.1
				NM_001341414.1
**BIP1/2** ^a^	AGGACTTTGACCACAGGATCA	TGCCCTCTCACATTCCCTTC	300	NM_122737.4
				NM_001344430.1
				NM_180788.3
				NM_123567.3
**BIP3** ^a^	CAAGGAACCCAGCAAAGGGA	GGCGCAACATCAAGCAGTAG	300	NM_001198015.2
				NM_100779.5
**PDI1** ^a^	CCACTACCGGAAAATAACGATGG	GGCCACACCAAGGAGCATA	300	NM_001125370.1
				NM_115353.5
**PDI5** ^a^	CATCCAACAAGGGACAGGGT	GTCCAAAGTACTGGAATGCACC	300	NM_102024.4
				NM_179365.1
**CNX1**	GGTCTCAAGAGCTACCAGAAGG	GTTTGGTTGTTGCTCGGCTT	300	NM_125573.4
**CNX2** ^a^	CCACACGAATCTTTTCAGTCCA	TCGCAAGTGAATTTGTTGTTGTT	300	NM_001336358.1
				NM_001036384.2
				NM_179846.4
				NM_001336359.1
				NM_001336357.1
**CRT1** ^a^	AAGCACAAGGATGCGGAGAA	CTTCCTCAGCGTCGGATTCA	300	NM_104513.5
				NM_001036122.2
**CRT2**	AAGCTCAAGGATGCGGAGAA	CAGCATCAGATTCCGCAGGT	300	NM_100791.4
**CRT3** ^a^	CCGGTATGGAGACAGGAGGA	GCCTCATAGCTCGTCATGGT	300	NM_100718.5
				NM_001198007.1
				NM_202064.1
**SYT1**	CGGTCAGAGATCCCCAGACT	TCTCGGGATTCCCAACCTGT	500	NM_127668.4
**VAP27-1**	AGAGACGGGGTGGAGAGAAT	AACTGCAACGTTCGTGGTTG	500	NM_115924.4
**NET3C**	GCATTGAGGTCTCCTTTGCG	TCCCTAACACAACAACATATCCCA	500	NM_001337284.1

a This primer pair amplifies every gene transcript variant.

b Pagliari et al. (2017).

## 3. Results

### 3.1. Phytoplasma infection induces conformational alterations of SE-ER, which may function as “docking berths” for phytoplasmas.

**Figure 1:**
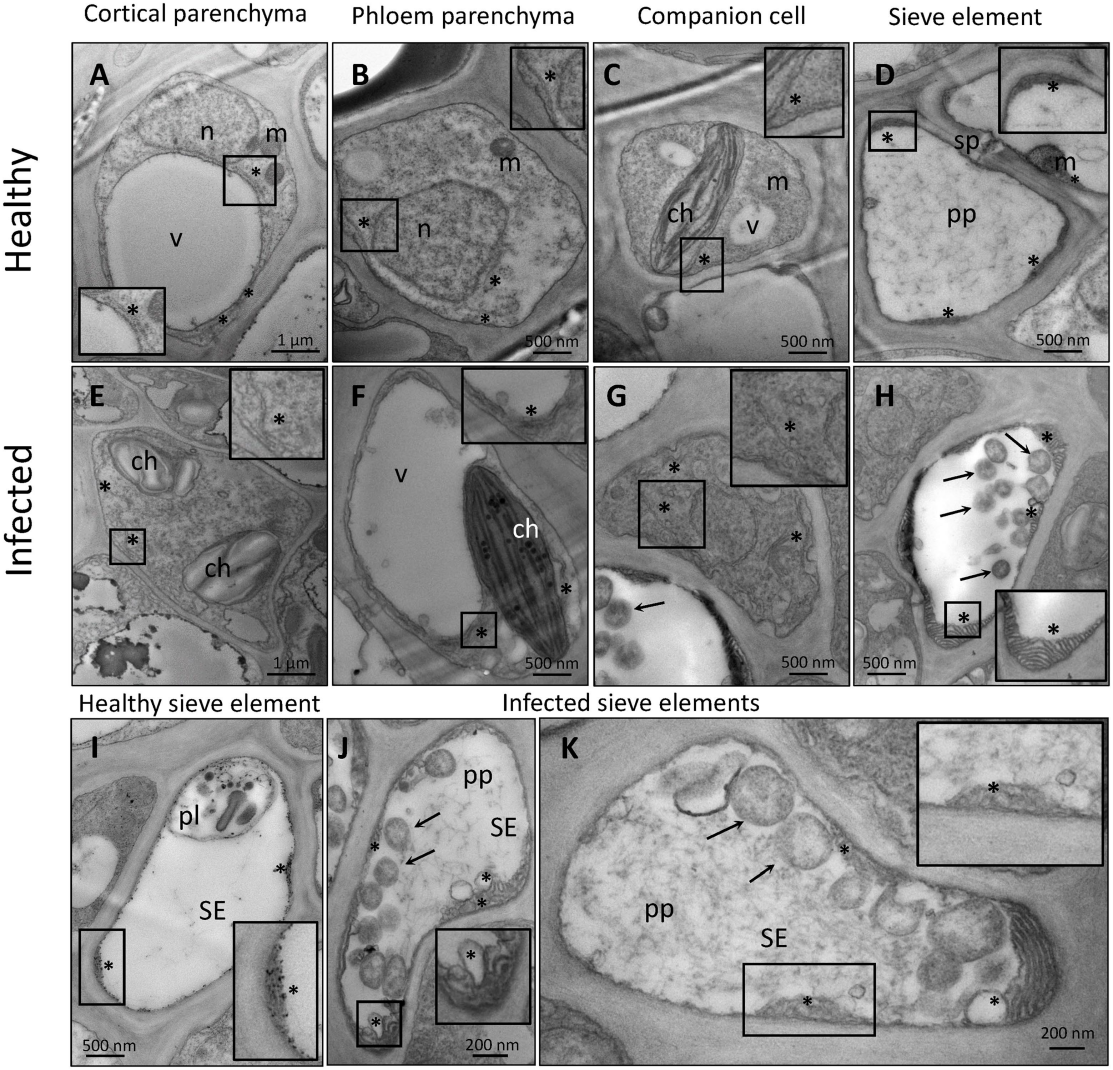
Representative TEM micrographs of healthy **(A–D,I)** and phytoplasma-infected **(E–H,J,K)** midrib cell types of Arabidopsis. In healthy midribs **(A–D,I)**, rough endoplasmic reticulum (r-ER) shows a conventional morphology in cortical parenchyma cells **(A)**, phloem parenchyma cells **(B)**, and companion cells **(C)**. The r-ER consists of regular tubules dotted with ribosomes, embedded in the cytosolic environment. In healthy sieve elements **(D,I)** sieve-element endoplasmic reticulum (SE-ER) stacks are free of ribosomes, firmly appressed in the cell perifery and mostly orientated in parallel to the plasma membrane. In infected midribs **(E–H, J,K)**, the r-ER does not show significant morphological alterations in the diverse cell types as compared to those in healthy plants **(E–G)**, with exception of the SE-ER in SEs **(H,J,K)**. The SE-ER shows dramatic conformational alterations such as increased number of stacks and the slackening or swelling of reticular cisternae **(H,J,K)**. In insets of **(A)**–**(K)** areas of interest are magnified. Arrows = phytoplasmas; asterisks: r-ER or SE-ER; ch: chloroplast; m: mitochondrion; n: nucleus; pl.: plastid; pp.: sieve-element protein; v: vacuole; SE: sieve element; sp.: sieve plate.

**Figure 2:**
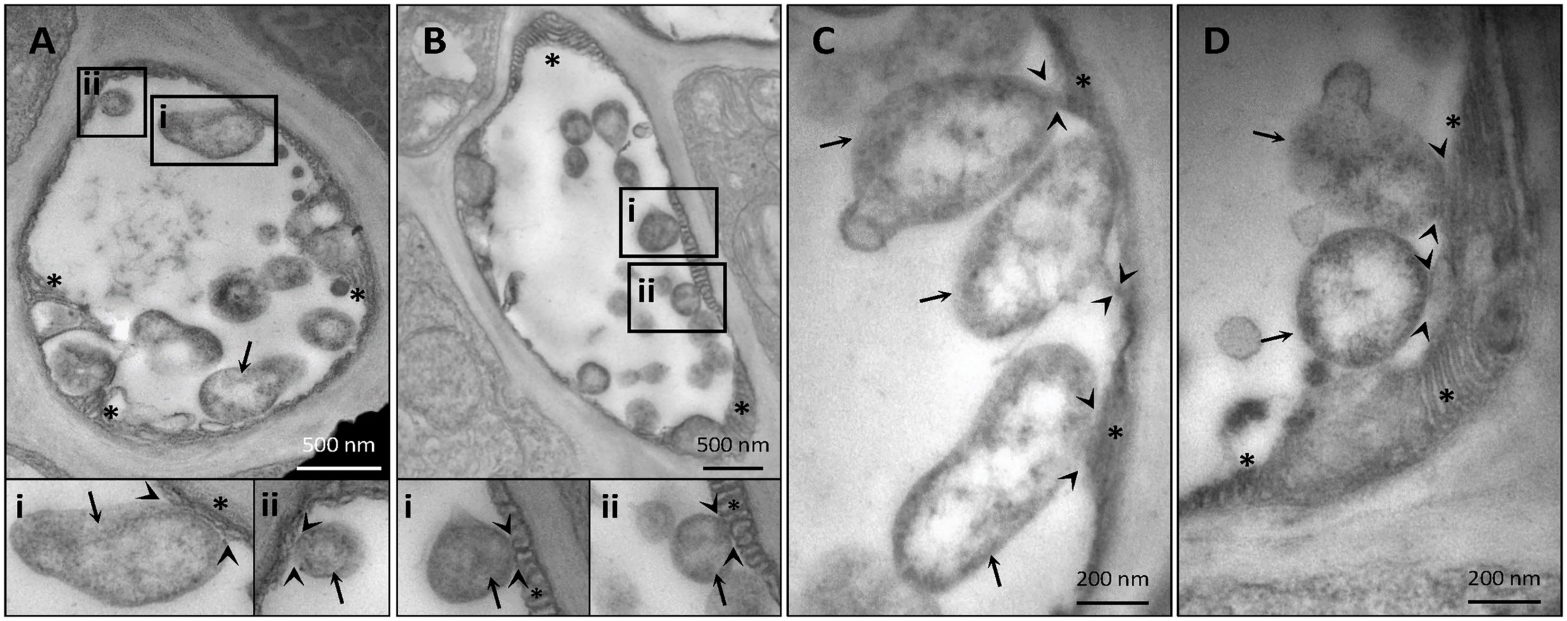
Representative TEM micrographs of infected sieve elements illustrate an adhesion of phytoplasmas to the sieve-element endoplasmic reticulum (SE-ER) **(A–D)**. The junction sites seldom show clear-cut outlines. In insets i and ii, areas of interest of **(A)** and **(B)** are magnified. Arrows = phytoplasmas; arrow-heads: phytoplasma-SE-ER contacts; asterisks: SE-ER; pp.: sieve-element protein.

Despite their restriction to SEs, phytoplasma can exert pathological effects throughout the whole plant *via* the secretion of effector proteins. Therefore, our TEM investigations on the effects of phytoplasma infection on SE-ER/r-ER morphology included diverse midrib cell types such as SEs, CCs, phloem parenchyma cells, and cortical parenchyma cells. In healthy plants, r-ER in cortical parenchyma, phloem parenchyma cells and CCs displayed a regular morphology in each cell-type examined (Figures 1A–C). Cortical parenchyma cells (Figure 1A), phloem parenchyma cells (Figure 1B), and CCs (Figure 1C) contained regular double-membrane ER-stacks heavily dotted with ribosomes, embedded in the cytosolic environment. In SEs of healthy plants, SE-ER stacks consisted of membranous stacks that were predominantly orientated in parallel to the plasma membrane, to which they were firmly appressed (Figures 1D,I). Phytoplasma infection did not lead to any significant alteration of ER organization of cortical parenchyma, phloem parenchyma cells or CCs (Figures 1E–G), but led to structural changes of the SE-ER (Figures 1H–K). There, infection induced dramatic conformational alterations such as an increased number of SE-ER stacks and swelling of the cisternae (Figures 1J,K). Phytoplasmas adhered to the malformed SE-ER membranes (Figure 2); the junction sites, however, did not reveal a precise and defined structure (Figures 2A–D).

### 3.2. Phytoplasma infection is associated with modulation of genes involved in reticulum stress responses.

**Figure 3:**
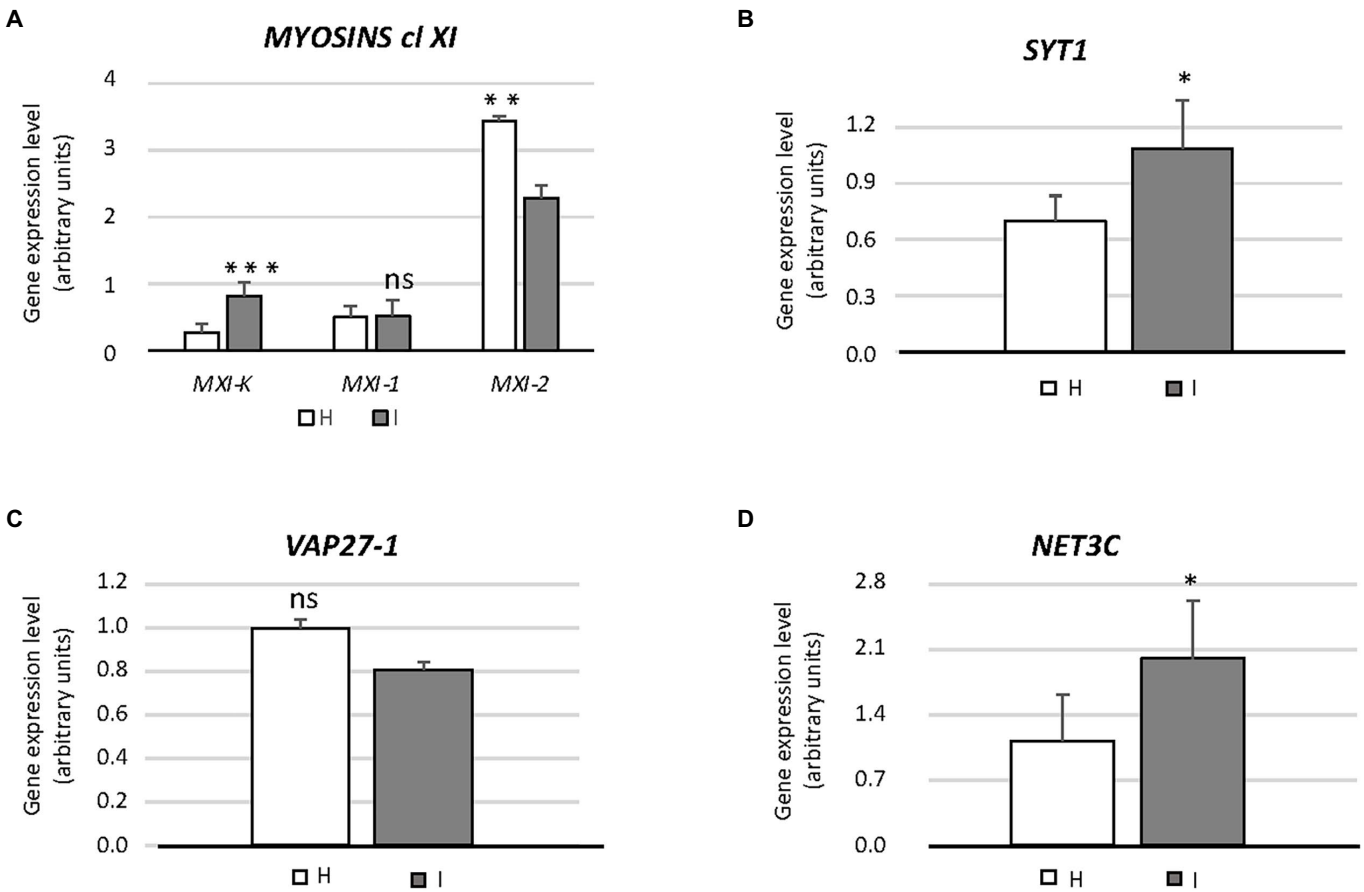
Expression of genes involved in endoplasmic reticulum architecture (*MXI-K*, *MXI-1*, *MXI-2*, *SYT1*, *VAP27-1*, *NET3C*) **(A–D)** in healthy and infected midrib tissue. Expression values were normalized to the *UBC9* transcript level, arbitrarily fixed at 100, then expressed as gene expression level (which corresponds to mean normalized expression, MNE) ± SD. At least five individuals (and three technical repeats of PCR reaction) were used for MNE determination of each gene in both healthy (H) and infected (I) plants. Statistical analyses were performed using an unpaired *t*-test. Family-wise significance and confidence level = 0.05 (*p < 0.05, **p < 0.01, ***p < 0.001, ns: not significant).

**Figure 4:**
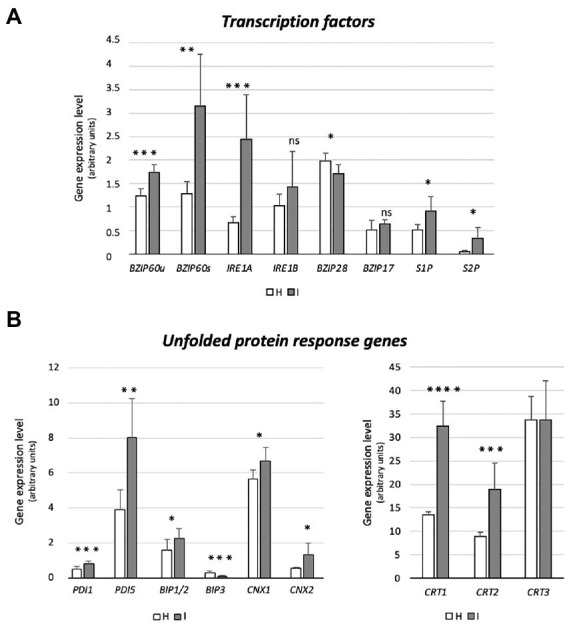
Expression of genes encoding UPR-promoting transcription factors (*bZIP60*, *IRE1a*, *IRE1b*, *bZIP17*, *bZIP28*, *S1P*, *S2P*) **(A)**, and UPR proteins (*BiP1/2*, *BIP3*, *CNX1*, *CNX2*, *CRT1*, *CRT2*, *CRT3*) **(B)** in healthy and infected midrib tissue. Expression values were normalized to the *UBC9* transcript level, arbitrarily fixed at 100, then expressed as gene expression level (which corresponds to mean normalized expression, MNE) ± SD. At least five individuals (and tree technical repeats of PCR reaction) were used for MNE determination of each gene, in both healthy (H) and infected (I) plants. Statistical analyses were performed using an unpaired t-test. Family-wise significance and confidence level = 0.05 (*p < 0.05, **p < 0.01, ***p < 0.001, ****p < 0.0001, ns: not significant).

The impact of phytoplasma infection on SE-ER/r-ER performance, was quantified by the expression level of genes, associated with ER functioning (Table 1), through real-time RT-PCR. Gene expression analyses are presented in Figures 3, 4, where the mean expression level of each gene is plotted as the transcript abundance normalized to the level of the internal control UBC9 (set at 100), in both healthy (H) and infected (I) plants.

The involvement of genes encoding class XI myosins in SE-ER re-shaping, in response to phytoplasma infection, was assessed using the transcript levels of *MXI-K*, *MXI-1* and *MXI-2* (Figure 3A). Phytoplasma infection modulated the expression of the three genes to a different degree. While the transcript level of *MXI* was more than doubled, transcription of *MXI-1* was not affected whereas *MXI-2* was downregulated to about 65% as compared to healthy plants (Figure 3A). Furthermore, we determined the expression levels of *SYT1*, *VAP27-1* and *NET3C,* presumably engaged in ER-PM anchoring or linking (Figures 3B–D). *SYT1* and *NET3C* were significantly overexpressed (*ca.* + 30 and + 50%, respectively) in case of phytoplasma infection (Figures 3B,D), whereas the expression of *VAP27-1* was the same in healthy and infected plants (Figure 3C).

Prior to the investigation of the expression levels of genes related to UPR, specific primers were designed to distinguish the *bZIP60* spliced (*bZIP60s*) and unspliced forms (*bZIP60u*), present in Arabidopsis (Figure 4A). Both forms were expressed to a significantly higher degree in response to phytoplasma infection (+41% and + 150%, respectively) (Figure 4A). Arabidopsis disposes of two genes with *IRE1*-related sequences, *IRE1*a and *IRE1*b. Phytoplasma infection caused a consistent increase of the *IRE1a* transcript level (+267%), but *IRE1b* expression was not changed (Figure 4A). While *bZIP28* expression was slightly downregulated (−15%), the transcription of the two genes encoding the proteins that activate bZIP28, S1P and S2P, were upregulated in reaction to infection (+77% and + 540%, respectively) (Figure 4A). Finally, the transcript level of another ER membrane-associated transcription factor, bZIP17 that is activated in a manner similar to bZIP28, remained stable (Figure 4A).

Phytoplasma infection induced the upregulation of *BiP1/2* transcripts, but led to *BiP3* downregulation (−60%) (Figure 4B). The Arabidopsis genome harbors 12 *PDI-like* genes, but only some of them are induced by the UPR. Among them, *PDI1* and *PDI5* were selected as representatives of this group. Transcription of both genes was significantly increased by phytoplasma infection (+60% for *PDI1* and + 106% for *PDI5*) (Figure 4B). Moreover, the transcription of two genes encoding CNXs in *A. thaliana*, *CNX1* and *CNX2,* were significantly upregulated by phytoplasma infection (roughly +20% and + 140%, respectively) (Figure 4B). Finally, three CRT genes, *CRT1*, *CRT2* and *CRT3* present in the Arabidopsis genome, infection increased *CRT1* and *CRT2* (+140% and + 111%) transcript levels, but not that of *CRT3* (Figure 4B).

## 4. Discussion

The r-ER displays a highly dynamic morphology to meet changing cellular requirements, including those associated with pathogenic attacks (Breeze et al., 2020; Jing and Wang, 2020). Similarly, SE-ER stacks frequently detached from the plasma membrane and fragmented into lobes and vesicles following phytoplasma infection (Buxa et al., 2015; Pagliari et al., 2016). These morphological changes are specific for the SE-ER in phytoplasma-infected SEs (Figures 1H–K). In contrast, the r-ER structure in neighboring cells (i. e. CCs, phloem parenchyma cells, or cortical parenchyma cells) remained unchanged during infection (Figures 1E–G). As a potential analogy, infection with a *Pseudomonas syringae* pv. *tomato*, strain DC3000, provoked a rapid r-ER remodelling in Arabidopsis leaf parenchyma cells, which solely occurred in cells bordering established bacterial colonies and was induced by effectors, but not by elicitors such as flagellin (Breeze et al., 2020).

Like other microbial pathogens, phytoplasmas secrete effectors that modulate both plant host and insect vector cell biology (Tomkins et al., 2018). The mode(s) of action exerted by phytoplasma effectors in the SE-ER modification is, however, unknown. Significant structural alterations and loss of viability of *Nicotiana benthamiana* protoplasts expressing the “*Candidatus* Phytoplasma mali” effector PME2_ST_ support the idea that phytoplasma effectors may primarily target plant cell membrane systems (Mittelberger et al., 2019). Effectors might also induce the formation of contact sites between phytoplasmas and SE-ER, as shown for numerous intracellular bacteria, docking on diverse host organelles (Dumox et al., 2012). Consistent with earlier work (Pagliari et al., 2016, 2017), the present observations (Figure 2) confirm phytoplasma anchoring of unknown identity to proximal SE-ER stacks. Such junctions may also serve as bridges for effector trafficking and pathways for host-resources supply (Tilney et al., 2001; Kagan et al., 2004).

**Figure 5:**
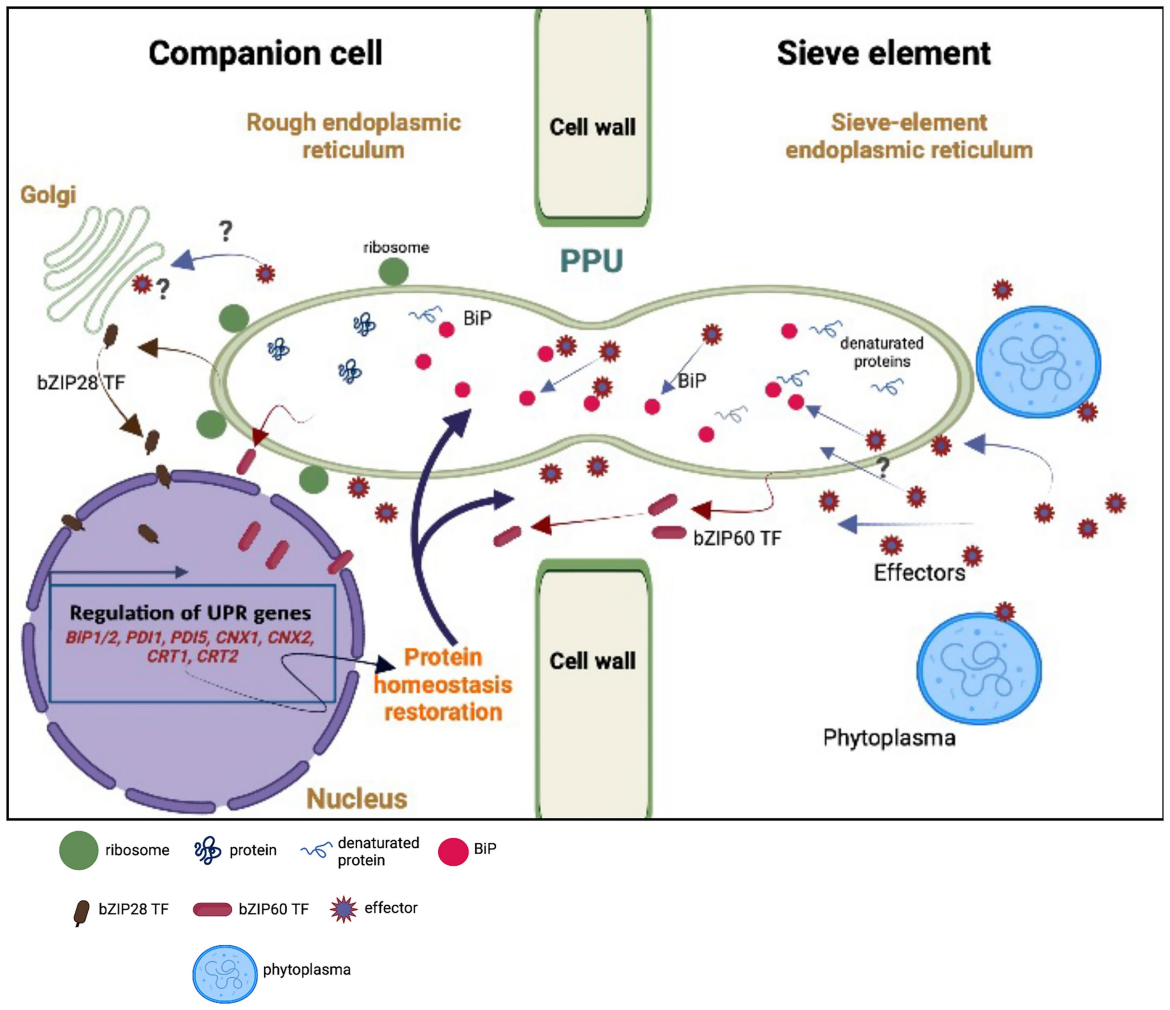
Model of hypothetical unfolded protein response (UPR) as the result of an interplay between the sieve element (SE) and the companion cell (CC). Upon perception of phytoplasma effectors, the sieve-element endoplasmic reticulum (SE-ER) is subject to stress (Buxa et al., 2015; Breeze et al., 2020) so that the number of misfolded proteins is rising due to the cleavage of BiPs. Stress sensing extends to the rough endoplasmic reticulum (r-ER) in the SE-adjacent cells (CCs, in particular) due to the effect of phytoplasma effectors (Bai et al., 2009) moving *via* cytosolic channels and/or r-ER/SE-ER connections in the pore-plasmodesma units (PPUs). Effectors are responsible for increased misfolding of proteins (Celli and Tsolis, 2015) and for the cleavage of BiPs, provoking the release of bZIP60 and bZIP28 transcription factors (TFs) in SEs and CCs. Local cleavage of BiPs in the SE-ER may confer diffusional withdrawal of BiPs from the r-ER in CCs *via* the connections between SE and SE-ER inside the PPUs. The question marks indicate uncertainties or missing knowledge on the putative events. The modified expression levels of the UPR-related genes must rely on the activities of the nucleate CCs or phloem parenchyma cells (PPCs; van Bel and Musetti, 2019) and UPR-associated proteins may traffic from the CCs to the SEs *via* both the r-ER-connections and the cytoplasmic corridors within the PPUs. For reasons of clarity, the structure of the PPUs does not represent the actual situation, in which the PPUs are branched at the CC-side.

However, the coarse approach applied - grinding entire midribs for gene-expression analysis - has an inevitable disadvantage, as it was not possible to assign infestation responses to specific cell types e.g. SEs or CCs. We could only sketch some tentative outlines of molecular SE-CC interaction, following a pathogenic attack (Figure 5).

We hypothesize that the presence of phytoplasmas is likely perceived by the SE-ER (Jing and Wang, 2020). However, it is unknown whether sensing is limited to the SE-ER, or if it also extends to the r-ER in SE-adjacent cells, in particular CCs (Figure 5), set in motion by phytoplasma effectors diffusing *via* plasmodesmata (Bai et al., 2009). Effectors may be responsible for an increased misfolding of proteins (Celli and Tsolis, 2015) and for partial cleavage of BiPs, as suggested by Jing and Wang (2020) (Figure 5). In addition, they could trigger bZIP-release from the dictyosomes in CCs (Figure 5). Moreover, bZIP messengers may be liberated in SEs and CCs due to cleavage of BiPs (Figure 5). On the other hand, local cleavage of BiPs in the SE-ER may confer diffusional loss of BiPs from the ER in CCs *via* the connection between SE and SE-ER inside the PPU (Figure 5). Amidst all these uncertainties regarding the location(s) of response, it is obvious that the enucleate SEs cannot be responsible for an increased or decreased gene expression, and that SE-responses to phytoplasma infection should primarily be investigated through CC-studies (van Bel and Musetti, 2019). Bearing this in mind, the question emerges whether UPR-related proteins move from CCs to SEs *via* cytoplasmic PPU channels or SE-ER/r-ER connecting PPU corridors (Figure 5).

To examine potential involvement of class XI myosin motor proteins (Griffing et al., 2014) in regulating SE-ER reorganization in response to phytoplasma infection, myosin gene expression was analyzed in healthy and phytoplasma-infected midrib tissues by real-time RT-PCR. We limited our analysis to genes coding for a subset of class XI myosins, i. e. MXI-K, MXI-1, and MXI-2, which are among the most highly expressed myosin isoforms in Arabidopsis (Ueda et al., 2010). *MXI-K* was significantly over-expressed in phytoplasma-infected midribs, whereas *MXI-1*, and *MXI-2* expression was not modulated or down-regulated, respectively (Figure 3A). The differential modulation of the myosin-encoding genes likely reflects their diverse functions. The r-ER-associated MXI-K is one of the chief controllers of reticulum movement and configuration (Ueda et al., 2010). MXI-K further modulates-likely indirectly–deposition of cellulose, callose, lignin-like compounds at infection sites (Ueda et al., 2010; Yang et al., 2014; Zhang et al., 2021), as described for other hosts responding to phytoplasma infection (Musetti et al., 2013; Buxa et al., 2015; De Marco et al., 2016; Pagliari et al., 2017).

MXI-1 and MXI-2 are effective in promoting plant growth (Duan et al., 2020). Their down-regulation (Figure 4A) is thus expected to concur with reduced host growth (Pagliari et al., 2017; Buoso et al., 2019; Bernardini et al., 2022). In addition to myosins XI, SYT1 is essential for maintaining the polygonal reticulum network in Arabidopsis. Moreover, SYT1 is vital for the stability/dynamics of other proteins, i.e., the VAP27-1/NET3C complexes that function as junctions at the ER-PM contact sites (Pérez-Sancho et al., 2015). Over-expression of *SYT1*, together with modulation of *VAP27-1* and/or *NET3C* would imply a drastic change in the number of ER-PM contact sites, in turn inducing r-ER deformation (Siao et al., 2016). Such a network malformation was found in response to abiotic stresses (Lee et al., 2019; Ruiz-Lopez et al., 2021) and also observed in phytoplasma-infected SE-ER (Figures 1H–J).

Apart from controlling r-ER network stability (Siao et al., 2016; Ishikawa et al., 2018) additional tasks have been attributed to SYT1, such as being involved in responses to wounding (Pérez-Sancho et al., 2015) and in the resistance to biotic challenges, such as viruses (Lewis and Lazarowitz, 2010; Uchiyama et al., 2014) and fungi (Kim et al., 2016). Apparently, SYT1 is also engaged in endocytotic processes, related to defense responses in Arabidopsis (Romanenko et al., 2002). Hence, increased *SYT1* expression levels (Figure 4B) could be related to the increased endocytic activity after phytoplasma attack, as demonstrated in Arabidopsis plants infected by the biotrophic fungus *Golovinomyces orontii* (Kim et al., 2016).

r-ER modifications, similar to those of the SE-ER configuration in infected midribs (Figures 2H–J), were described to reflect the UPR (Bernales et al., 2006; Choi and Song, 2020 and literature therein). In phytoplasma-infected *Paulownia* plants, genes encoding UPR-related proteins were among the top 20 differentially expressed (Mou et al., 2013). Regulation of genes associated with UPR in response to phytoplasma infection has not yet been investigated. *IRE1a* is over-expressed in infected plants (Figure 4A), which seems a general response to biotic stresses (Moreno et al., 2012). By contrast, expression of *IRE1b*, required to activate cell autophagy in response to persistent stress (Bao et al., 2018), is not modulated (Figure 4A), consistent with the notion that phytoplasmas must save host-cell viability to ensure their own survival and propagation (van Bel and Musetti, 2019). Concomitant non- or down-regulation of *bZIP17/28* in response to phytoplasmas indicates complex interactions between several UPR-related TFs (Gayral et al., 2020; Pastor-Cantizano et al., 2020). Overlapping functions of bZIP60 and bZIP17/28 TFs, for which a precise extent is yet to be determined may provide an explanation for the discontinuity as well as the modulation of the targeting process (Ruberti et al., 2015). Therefore, it has to be defined whether activation of bZIP28 and bZIP60 occurs simultaneously or sequentially, at the onset of the reticulum stress.

The genes encoding S1P and S2P proteins, located in Golgi bodies, absent in SEs, were modulated in phytoplasma-infected midribs (Figure 5). *S1P* and *S2P* were up-regulated in infected samples, suggesting that transcriptional induction of proteases (i. e. S1P and S2P) might precede the induction of the target proteins during UPR (i. e. bZIP28; Vitale et al., 2015). S1P and S2P are involved in pathways related to hormone signaling (Zhou et al., 2015), which are strongly affected by phytoplasma attacks (Dermastia, 2019; Bernardini et al., 2020).

Phytoplasmas trigger UPR, that affects the transcription of ER-quality control compounds. After being activated, TFs reach the nucleus and modulate the expression of a set of stress-responsive genes (Figure 5), including those coding for BiP, CNX, CRT, and PDI (Nawkar et al., 2018). In general, UPR-associated genes are upregulated in response to phytoplasma infection (Figures 3, 4), which demonstrates the need of proper protein folding, that may become unbalanced by phytoplasma proliferation.

While sequences and folding structures are highly similar, the three BiP genes of Arabidopsis are readily distinguishable by their expression in different tissues (Reyes-Impellizzeri and Moreno, 2021), in response to distinct stimuli (Herath et al., 2020). *BiP1* and *BiP2* are nearly identical (99% protein identity), and seem to have partly overlapping functions (Herath et al., 2020 and literature therein). They are constitutively expressed and linked to developmental processes, whereas *BiP3* is exclusively expressed in reaction to stress (Herath et al., 2020). As for plant/pathogen interactions, modulation of BiP encoding genes serves reticulum stress-protective mechanisms, as well as the regulation of plant immune responses (Moon and Park, 2016).

An increase in BiP2 transcripts is likely required to induce pathogenesis-related (PR) protein synthesis and to promote systemic acquired resistance (SAR). Knocking out *BiP2* provokes compromised secretion of PR1 and enhanced colonization by viral, bacterial and fungal pathogens (Verchot and Pajerowska-Mukhtar, 2021). Upon virus infection, overexpression of *BiP2* also suppresses imminent cell-death symptoms, caused by r-ER collapse (Pagliari et al., 2021; Verchot and Pajerowska-Mukhtar, 2021). Thus, the phytoplasma-elicited overexpression of *BiP1/2* (Figure 4B) is not only related to an induction of a reticulum-stress response (Lu and Christopher, 2008), but is also an attempt to prevent host cell death. Phytoplasma-induced *BIP1/2* expression is thus consistent with a simultaneous down-regulation of cell-death promoting *BiP3* (Figure 4B; Moon and Park, 2016). A lower expression level of *BiP3* upon phytoplasma infection (Figure 4B) also coincides with a reduction of *bZIP28* transcripts (Figure 4A).

Modulation of other UPR-associated marker genes (i. e. *PDI*, *CNX*, *CRT*) further indicates further phytoplasma-triggered reticulum stress. The 12 PDI genes of *A. thaliana* encode signal proteins, some of which (i. e. PDI 1 and 5) possess an ER retention signal (KDEL sequence) at the carboxyl terminus (Alanen et al., 2003) and display chaperone activities, helping correct protein folding/unfolding (Strasser, 2018). Arabidopsis mutants silenced for *PDI1* showed an increased sensitivity to stress, whereas plant lines overexpressing *PDI1* exhibited increased tolerance, which suggests that PDI1 has a role in stress mitigation (Zhang et al., 2018). The genes encoding CNXs and CRTs were up-regulated upon phytoplasma infection, with exception of *CRT3*, which was not modulated. The over-expression is indicative of enhanced activity of the protein folding machinery (Liu and Howell, 2016). CNXs are central elements of the ER-quality control system for N-glycoproteins in eukaryotic cells. Interestingly, a role of protein N-glycosylation in defensive responses to bacterial infection has been described for Arabidopsis (Gao et al., 2022).

CRT1/CRT2 have a unique function as key alleviators of ER stress in plants (Qiu et al., 2012). Furthermore, CRT2 is considered an important player in the Arabidopsis immune response, as it may promote or suppress the plant defense reaction, by a self-regulatory activity (Qiu et al., 2012). This is a finely-tuned mechanism, which favors, for example, the infection process of the biotrophic pathogen *Pseudomonas syringae* pv*. tomato* DC3000, by limiting the salicylic acid-mediated plant defense response (Qiu et al., 2012). A similar mechanism could be elicited by phytoplasmas to ensure their own survival (Bernardini et al., 2020). Expression of *CRT3* was not modified in Arabidopsis by phytoplasma infection, because it may be specifically required for biogenesis of the EF-Tu (elongation factor-thermo unstable) receptor, associated with its responsiveness to bacterial pathogen-associated molecular patterns (Li et al., 2009).

## 5. Concluding remarks

Phytoplasma infection induces (1) restructuring and reorientation of the SE-ER; (2) differential expression of genes encoding proteins involved in shaping and anchoring the SE-ER; (3) increased release of bZIP60 signals from the SE-ER/r-ER stacks; (4) massive changes in the expression of UPR-associated genes that likely reflect a trade-off between survival of host cells, needed for the phytoplasmic biotrophic lifestyle, and phytoplasmas.

In conclusion, UPR must be considered as part of the phloem-based immune reaction induced by phytoplasma infection. It should be stressed once again that the modified expression levels likely rely on the activities of the nucleate CCs (van Bel and Musetti, 2019) and that trafficking of proteins from and to SEs depends on the SE-ER/r-ER corridor and/or cytoplasmic channels within PPUs (Figure 5).

## Key contribution

Phytoplasmas modulate sieve-element endoplasmic reticulum structure and function in such a manner that their biotrophic lifestyle is sustained, while the host cells remain viable.

## Data availability statement

The original contributions presented in the study are included in the article/Supplementary material, further inquiries can be directed to the corresponding author.

## Author contributions

RM: conceptualization, supervision, project administration, and funding acquisition. RM, LP, SS, CB, GM, and FC: methodology. RM, LP, CB, and SS: investigation. RM and SS: data curation. RM, LP, and AvB: writing-original draft preparation. RM, SS, and AvB: writing-review and editing. All authors have read and agreed to the published version of the manuscript.

## Funding

Open access funding provided by the Department of Agricultural, Food, Environmental and Animal Sciences, University of Udine.

## Conflict of interest

The authors declare that the research was conducted in the absence of any commercial or financial relationships that could be construed as a potential conflict of interest.

## Publisher’s note

All claims expressed in this article are solely those of the authors and do not necessarily represent those of their affiliated organizations, or those of the publisher, the editors and the reviewers. Any product that may be evaluated in this article, or claim that may be made by its manufacturer, is not guaranteed or endorsed by the publisher.

## References

[ref1] Adams C. J. Kopp M. C. Larburu N. Nowak P. R. Ali M. M. U. (2019). Structure and molecular mechanism of ER stress signaling by the unfolded protein response signal activator IRE1. Front. Mol. Biosci. 6:11. doi: 10.3389/fmolb.2019.0001, PMID: 30931312 PMC6423427

[ref2] Alanen H. I. Williamson R. A. Howard M. J. Lappi A. K. Jäntti H. P. Rautio S. M.. (2003). Functional characterization of ERp18, a new endoplasmic reticulum-located thioredoxin superfamily member. J. Biol. Chem. 278, 28912–28920. doi: 10.1074/jbc.M304598200, PMID: 12761212

[ref3] Almeida C. (2021). A potential third-order role of the host endoplasmic reticulum as a contact site in interkingdom microbial endosymbiosis and viral infection. Environ. Microbiol. Rep. 13, 255–271. doi: 10.1111/1758-2229.12938, PMID: 33559322

[ref4] Bai X. Correa V. R. Toruño T. Y. Ammar E. D. Kamoun S. Hogenhout S. A. (2009). AY-WB phytoplasma secretes a protein that targets plant cell nuclei. Mol. Plant-Microbe Interact. 22, 18–30. doi: 10.1094/MPMI-22-1-0018, PMID: 19061399

[ref5] Bao Y. Bassham D. C. Howell S. H. (2019). A functional unfolded protein response is required for normal vegetative development. Plant Physiol. 179, 1834–1843. doi: 10.1104/pp.18.01261, PMID: 30710050 PMC6446744

[ref6] Bao Y. Pu Y. Yu X. Gregory B. D. Srivastava R. Howell S. H.. (2018). IRE1B degrades RNAs encoding proteins that interfere with the induction of autophagy by ER stress in *Arabidopsis thaliana*. Autophagy 14, 1562–1573. doi: 10.1080/15548627.2018.1462426, PMID: 29940799 PMC6135571

[ref7] Bernales S. McDonald K. L. Walter P. (2006). Autophagy counterbalances endoplasmic reticulum expansion during the unfolded protein response. PLoS Biol. 4:e423. doi: 10.1371/journal.pbio.0040423, PMID: 17132049 PMC1661684

[ref8] Bernardini C. Pagliari L. De Rosa V. Almeida-Trapp M. Santi S. Martini M.. (2020). Pre-symptomatic modified phytohormone profile is associated with lower phytoplasma titres in an *Arabidopsis* seor1ko line. Sci. Rep. 10:14770. doi: 10.1038/s41598-020-71660-0, PMID: 32901060 PMC7479616

[ref9] Bernardini C. Santi S. Mian G. Levy A. Buoso S. Suh J. H.. (2022). Increased susceptibility to chrysanthemum yellows phytoplasma infection in *Atcals7ko* plants is accompanied by enhanced expression of carbohydrate transporters. Planta 256:43. doi: 10.1007/s00425-022-03954-8, PMID: 35842878 PMC9288947

[ref10] Bosco D. Minucci C. Boccardo G. Conti M. (1997). Differential acquisition of chrysanthemum yellows phytoplasma by three leafhopper species. Entomol. Exp. Appl. 83, 219–224. doi: 10.1046/j.1570-7458.1997.00175.x

[ref11] Breeze E. Vale V. McLellan H. Godiard L. Grant M. Frigerio L. (2020). The plant endoplasmic reticulum is both receptive and responsive to pathogen effectors. bioRxiv 2020.06.09.142141. doi: 10.1101/2020.06.09.142141

[ref12] Buoso S. Pagliari L. Musetti R. Martini M. Marroni F. Schmidt W.. (2019). ‘*Candidatus* Phytoplasma solani’ interferes with the distribution and uptake of iron in tomato. BMC Genomics 20:703. doi: 10.1186/s12864-019-6062-x, PMID: 31500568 PMC6734453

[ref13] Buxa S. V. Degola F. Polizzotto R. De Marco F. Loschi A. Kogel K. H.. (2015). Phytoplasma infection in tomato is associated with re-organization of plasma membrane, ER stacks, and actin filaments in sieve elements. Front. Plant Sci. 6:650. doi: 10.3389/fpls.2015.00650, PMID: 26347766 PMC4541602

[ref14] Celli J. Tsolis R. M. (2015). Bacteria, the ER and the unfolded protein response: friends or foes? Nat. Rev. Microbiol. 13, 71–82. doi: 10.1038/nrmicro3393, PMID: 25534809 PMC4447104

[ref16] Choi J. A. Song C. H. (2020). Insights into the role of endoplasmic reticulum stress in infectious diseases. Front. Immunol. 10:3147. doi: 10.3389/fimmu.2019.0314, PMID: 32082307 PMC7005066

[ref17] De Marco F. Pagliari L. Degola F. Buxa S. V. Loschi A. Dinant S.. (2016). Combined microscopy and molecular analyses show phloem occlusions and cell wall modifications in tomato leaves in response to ‘*Candidatus* Phytoplasma solani. J. Microsc. 263, 212–225. doi: 10.1111/jmi.12426 27197728

[ref18] Dermastia M. (2019). Plant hormones in phytoplasma infected plants. Front. Plant Sci. 10:477. doi: 10.3389/fpls.2019.00477, PMID: 31057582 PMC6478762

[ref19] Duan Z. Ito K. Tominaga M. (2020). Heterologous transformation of *Camelina sativa* with high-speed chimeric myosin XI-2 promotes plant growth and leads to increased seed yield. Plant Biotechnol. 37, 253–259. doi: 10.5511/plantbiotechnology.20.0225b, PMID: 33088188 PMC7557661

[ref20] Dumox M. Clare D. K. Saibil H. R. Hayward R. D. (2012). *Chlamydiae* assemble a pathogen synapse to hijack the host endoplasmic reticulum. Traffic 13, 1612–1627. doi: 10.1111/tra.12002, PMID: 22901061 PMC3533787

[ref21] Ehlers K. Knoblauch M. van Bel A. J. E. (2000). Ultrastructural features of well-preserved and injured sieve elements: minute clamps keep the phloem transport conduits free for mass flow. Protoplasma 214, 80–92. doi: 10.1007/BF02524265

[ref22] Gao H. Ma K. Ji G. Pan L. Wang Z. Cui M.. (2022). Protein glycosylation changes during systemic acquired resistance in *Arabidopsis thaliana*. Int. J. Biol. Macromol. 212, 381–392. doi: 10.1016/j.ijbiomac.2022.05.126, PMID: 35623457

[ref23] Gayral M. Arias Gaguancela O. Vasquez E. Herath V. Flores F. J. Dickman M. B.. (2020). Multiple ER-to-nucleus stress signalling pathways are activated during *Plantago asiatica* mosaic virus and turnip mosaic virus infection in *Arabidopsis thaliana*. Plant J. 103, 1233–1245. doi: 10.1111/tpj.14798, PMID: 32390256

[ref24] Griffing L. R. Gao H. T. Sparkes I. (2014). ER network dynamics are differentially controlled by myosins XI-K, XI-C, XI-E, XI-I, XI-1, and XI-2. Front. Plant Sci. 5:218. doi: 10.3389/fpls.2014.00218, PMID: 24904614 PMC4033215

[ref25] Herath V. Gayral M. Adhikari N. Miller R. Verchot J. (2020). Genome-wide identification and characterization of *Solanum tuberosum* BiP genes reveal the role of the promoter architecture in BiP gene diversity. Sci. Rep. 10:11327. doi: 10.1038/s41598-020-68407-2, PMID: 32647371 PMC7347581

[ref28] Howell S. H. (2021). Evolution of the unfolded protein response in plants. Plant Cell Environ. 44, 2625–2635. doi: 10.1111/pce.14063, PMID: 33840122

[ref30] Ishikawa K. Tamura K. Ueda H. Ito Y. Nakano A. Hara-Nishimura I.. (2018). Synaptotagmin-associated endoplasmic reticulum-plasma membrane contact sites are localized to immobile ER tubules. Plant Physiol. 178, 641–653. doi: 10.1104/pp.18.00498, PMID: 30126867 PMC6181054

[ref001] Iwata Y. Koizumi N. (2012). Plant transducers of the endoplasmic reticulum unfolded protein response. Trends Plant Sci. 17, 720–727. doi: 10.1016/j.tplants.2012.06.014 22796463

[ref31] Jiang Y. Zhange C.-X. Chen R. He S. Y. (2019). Challenging battles of plants with phloem-feeding insects and prokaryotic pathogens. Proc. Natl. Acad. Sci. U. S. A. 116, 23390–23397. doi: 10.1073/pnas.1915396116, PMID: 31712429 PMC6876188

[ref32] Jing M. Wang Y. (2020). Plant pathogens utilize effectors to hijack the host endoplasmic reticulum as part of their infection strategy. Engineering 6, 500–504. doi: 10.1016/j.eng.2020.03.003

[ref33] Kagan J. C. Stein M. P. Pypaert M. Roy C. R. (2004). *Legionella* subvert the functions of Rab1 and Sec22b to create a replicative organelle. J. Exp. Med. 199, 1201–1211. doi: 10.1084/jem.20031706, PMID: 15117975 PMC2211909

[ref34] Kim K. T. Jeon J. Choi J. Cheong K. Song H. Choi G.. (2016). Kingdom-wide analysis of fungal small secreted proteins (SSPs) reveals their potential role in host association. Front. Plant Sci. 7:186. doi: 10.3389/fpls.2016.00186, PMID: 26925088 PMC4759460

[ref35] Kloth K. J. Shah P. Broekgaarden C. Ström C. Albrectsen B. R. Dicke M. (2021). SLI1 confers broad-spectrum resistance to phloem-feeding insects. Plant Cell Environ. 44, 2765–2776. doi: 10.1111/pce.14064, PMID: 33837973 PMC8360143

[ref36] Koizumi N. (1996). Isolation and responses to stress of a gene that encodes a luminal binding protein in *Arabidopsis thaliana*. Plant Cell Physiol. 37, 862–865. doi: 10.1093/oxfordjournals.pcp.a029023, PMID: 8888624

[ref38] Kriechbaumer V. Brandizzi F. (2020). The plant endoplasmic reticulum: an organized chaos of tubules and sheets with multiple functions. J. Microsc. 280, 122–133. doi: 10.1111/jmi.12909, PMID: 32426862 PMC10895883

[ref39] Kube M. Mitrovic J. Duduk B. Rabus R. Seemüller E. (2012). Current view on phytoplasma genomes and encoded metabolism. Sci. World J. 2012:185942. doi: 10.1100/2012/185942, PMID: 22550465 PMC3322544

[ref40] Lee I. M. Martini M. Marcone C. Zhu S. F. Y. (2004). Classification of phytoplasma strains in the elm yellows group (16SrV) and proposal of ‘*Candidatus* Phytoplasma ulmi’ for the phytoplasma associated with elm yellows. Int. J. Syst. Evol. Microbiol. 54, 337–347. doi: 10.1099/ijs.0.02697-0, PMID: 15023941

[ref41] Lee E. Vanneste S. Pérez-Sancho J. Benitez-Fuente F. Strelau M. Macho A. P.. (2019). Ionic stress enhances ER–PM connectivity via phosphoinositide-associated SYT1 contact site expansion in *Arabidopsis*. Proc. Natl. Acad. Sci. U. S. A. 116, 1420–1429. doi: 10.1073/pnas.1818099116, PMID: 30610176 PMC6347719

[ref42] Lewis J. D. Knoblauch M. Turgeon R. (2022). The phloem as an arena for plant pathogens. Annu. Rev. Phytopathol. 60, 77–96. doi: 10.1146/annurev-phyto-020620-100946, PMID: 35385671

[ref43] Lewis J. D. Lazarowitz S. G. (2010). Arabidopsis synaptotagmin SYTA regulates endocytosis and virus movement protein cell-to-cell transport. Proc. Natl. Acad. Sci. U. S. A. 107, 2491–2496. doi: 10.1073/pnas.0909080107, PMID: 20133785 PMC2823903

[ref44] Li W. Abad J. A. French-Monar R. D. Rascoe J. Wen A. Gudmestad N. C.. (2009). Multiplex real-time PCR for detection, identification and quantification of ‘*Candidatus* Liberibacter solanacearum’ in potato plants with zebra chip. J. Microbiol. Meth. 78, 59–65. doi: 10.1016/j.mimet.2009.04.009, PMID: 19409423

[ref45] Liu J. X. Howell S. H. (2016). Managing the protein folding demands in the endoplasmic reticulum of plants. New Phytol. 211, 418–428. doi: 10.1111/nph.13915, PMID: 26990454

[ref46] Liu J. X. Srivastava R. Che P. Howell S. H. (2007). An endoplasmic reticulum stress response in *Arabidopsis* is mediated by proteolytic processing and nuclear relocation of a membrane-associated transcription factor, bZIP28. Plant Cell 19, 4111–4119. doi: 10.1105/tpc.106.050021, PMID: 18156219 PMC2217655

[ref47] Liu Y. Vasina V. V. Kraner M. E. Peters W. S. Sonnewald U. Knoblauch M. (2022). Proteomics of isolated sieve tubes from *Nicotiana tabacum*: sieve element–specific proteins reveal differentiation of the endomembrane system. Proc. Natl. Acad. Sci. U. S. A. 119:e2112755119. doi: 10.1073/pnas.2112755119, PMID: 34983847 PMC8740716

[ref48] Lu D. P. Christopher D. A. (2008). Endoplasmic reticulum stress activates the expression of a sub-group of protein disulphide isomerase genes and AtbZIP60 modulates the response in *Arabidopsis thaliana*. Mol. Genet. Genomics 280, 199–210. doi: 10.1007/s00438-008-0356-z, PMID: 18574595

[ref49] Mapuranga J. Zhang N. Zhang L. Chang J. Yang W. (2022). Infection strategies and pathogenicity of biotrophic plant fungal pathogens. Front. Microbiol. 13:799396. doi: 10.3389/fmicb.2022.799396, PMID: 35722337 PMC9201565

[ref50] Martens H. Roberts A. G. Oparka K. J. Schulz A. (2006). Quantification of plasmodesmatal endoplasmic reticulum coupling between sieve elements and companion cells using fluorescence redistribution after photobleaching. Plant Physiol. 142, 471–480. doi: 10.1104/pp.106.085803, PMID: 16905664 PMC1586037

[ref51] Mittelberger C. Stellmach H. Hause B. Kerschbamer C. Schlink K. Letschka T.. (2019). A novel effector protein of apple proliferation phytoplasma disrupts cell integrity of *nicotiana* spp. protoplasts. Int. J. Mol. Sci. 20:4613. doi: 10.3390/ijms20184613, PMID: 31540359 PMC6770106

[ref52] Moon J. Y. Park J. M. (2016). Cross-talk in viral defense signaling in plants. Front. Microbiol. 7:2068. doi: 10.3389/fmicb.2016.02068, PMID: 28066385 PMC5174109

[ref53] Moreno A. A. Mukhtar M. S. Blanco F. Boatwright J. L. Moreno I. Jordan M. R.. (2012). IRE1/bZIP60-mediated unfolded protein response plays distinct roles in plant immunity and abiotic stress responses. PLoS One 7:e31944. doi: 10.1371/journal.pone.0031944, PMID: 22359644 PMC3281089

[ref54] Mou H. Q. Lu J. Zhu S. F. Lin C. L. Tian G.-Z. Xu X.. (2013). Transcriptomic analysis of *paulownia* infected by paulownia witches’-broom phytoplasma. PLoS One 8:e77217. doi: 10.1371/journal.pone.0077217, PMID: 24130859 PMC3795066

[ref55] Muller P. Y. Janovjak H. Miserez A. R. Dobbie Z. (2002). Processing of gene expression data generated by quantitative real-time RT-PCR. BioTechniques 32:6. ISSN: 1372-1379 12074169

[ref56] Musetti R. Buxa S. V. De Marco F. Loschi A. Polizzotto R. Kogel K.-H.. (2013). Phytoplasma-triggered ca ^2+^ influx is involved in sieve-tube blockage. Mol. Plant-Microbe Interact. 26, 379–386. doi: 10.1094/MPMI-08-12-0207-R, PMID: 23234405

[ref57] Namba S. (2019). Molecular and biological properties of phytoplasmas. Proc. Jpn. Acad. Ser. B 95, 401–418. doi: 10.2183/pjab.95.028, PMID: 31406061 PMC6766451

[ref58] Nawkar G. M. Lee E. S. Shelake R. M. Park J. H. Ryu S. W. Kang C. H.. (2018). Activation of the transducers of unfolded protein response in plants. Front. Plant Sci. 9:214. doi: 10.3389/fpls.2018.00214, PMID: 29515614 PMC5826264

[ref59] Nelson D. E. Glaunsinger B. Bohnert H. J. (1997). Abundant accumulation of the calcium-binding molecular chaperone calreticulin in specific floral tissues of *Arabidopsis thaliana*. Plant Physiol. 114, 29–37. doi: 10.1104/pp.114.1.29, PMID: 9159940 PMC158275

[ref60] Oparka K. J. Johnson R. P. C. Bowen I. D. (1981). Sites of acid phosphatase in the differentiating root protophloem of *Nymphoides peltata* (SG Gmel.) O. Kuntze. Support for the role of stacked ER in sieve-element autolysis. Plant Cell Environ. 4, 27–35. doi: 10.1111/j.1365-3040.1981.tb00832.x

[ref61] Oshima Y. Shikata M. Koyama T. Ohtsubo N. Mitsuda N. Ohme-Takagi M. (2013). MIXTA-like transcription factors and WAX INDUCER1/SHINE1 coordinately regulate cuticle development in *Arabidopsis* and *Torenia fournieri*. Plant Cell 25, 1609–1624. doi: 10.1105/tpc.113.110783, PMID: 23709630 PMC3694695

[ref62] Pagliari L. Buoso S. Santi S. Furch A. C. U. Martini M. Degola F.. (2017). Filamentous sieve element proteins are able to limit phloem mass flow, but not phytoplasma spread. J. Exp. Bot. 68, 3673–3688. doi: 10.1093/jxb/erx199, PMID: 28859375 PMC5853782

[ref63] Pagliari L. Martini M. Loschi A. Musetti R. (2016). Looking inside phytoplasma-infected sieve elements: a combined microscopy approach using *Arabidopsis thaliana* as a model plant. Micron 89, 87–97. doi: 10.1016/j.micron.2016.07.007, PMID: 27569416

[ref64] Pagliari L. Tarquini G. Loschi A. Buoso S. Kapun G. Ermacora P.. (2021). Gimme shelter: three-dimensional architecture of the endoplasmic reticulum, the replication site of grapevine pinot gris virus. Funct. Plant Biol. 48, 1074–1085. doi: 10.1071/FP21084, PMID: 34462050

[ref65] Park C. J. Park J. M. (2019). Endoplasmic reticulum plays a critical role in integrating signals generated by both biotic and abiotic stress in plants. Front. Plant Sci. 10:399. doi: 10.3389/fpls.2019.00399, PMID: 31019523 PMC6458287

[ref66] Pastor-Cantizano N. Ko D. K. Angelos E. Pu Y. Brandizzi F. (2020). Functional diversification of ER stress responses in Arabidopsis. Trends Biochem. Sci. 45, 123–136. doi: 10.1016/j.tibs.2019.10.008, PMID: 31753702 PMC6980780

[ref67] Pérez-Sancho J. Vanneste S. Lee E. McFarlane H. E. Esteban del Valle A. Valpuesta V.. (2015). The Arabidopsis synaptotagmin1 is enriched in endoplasmic reticulum-plasma membrane contact sites and confers cellular resistance to mechanical stresses. Plant Physiol. 168, 132–143. doi: 10.1104/pp.15.00260, PMID: 25792253 PMC4424031

[ref68] Pfaffl M. W. (2001). A new mathematical model for relative quantification in real-time RT–PCR. Nucl. Ac. Res. 29, e45–e445. doi: 10.1093/nar/29.9.e45, PMID: 11328886 PMC55695

[ref69] Qiu Y. Xi J. Du L. Roje S. Poovaiah B. W. (2012). A dual regulatory role of *Arabidopsis* calreticulin-2 in plant innate immunity. Plant J. 69, 489–500. doi: 10.1111/j.1365-313X.2011.04807.x, PMID: 21974727

[ref70] Reyes-Impellizzeri S. Moreno A. A. (2021). The endoplasmic reticulum role in the plant response to abiotic stress. Front. Plant Sci. 12:755447. doi: 10.3389/fpls.2021.755447, PMID: 34868142 PMC8637532

[ref71] Romanenko A. S. Rifel A. A. Salyaev the Corresponding Member of the RAS R. K (2002). Endocytosis of exopolysaccharides of the potato ring rot causal agent by host-plant cells. Doklady Biolog. Sci. 386, 451–453. doi: 10.1023/A:1020774503820, PMID: 12469411

[ref72] Ruberti C. Kim S. J. Stefano G. Brandizzi F. (2015). Unfolded protein response in plants: one master, many questions. Curr. Opin. Plant Biol. 27, 59–66. doi: 10.1016/j.pbi.2015.05.016, PMID: 26149756 PMC4618186

[ref73] Ruiz-Lopez N. Pérez-Sancho J. del Valle A. E. Haslam R. P. Vanneste S. Catalá R.. (2021). Synaptotagmins at the endoplasmic reticulum–plasma membrane contact sites maintain diacylglycerol homeostasis during abiotic stress. Plant Cell 33, 2431–2453. doi: 10.1093/plcell/koab122, PMID: 33944955 PMC8364230

[ref74] Schapire A. L. Voigt B. Jasik J. Rosado A. Lopez-Cobollo R. Menzel D.. (2008). *Arabidopsis* Synaptotagmin 1 is required for the maintenance of plasma membrane integrity and cell viability. Plant Cell 20, 3374–3388. doi: 10.1105/tpc.108.063859, PMID: 19088329 PMC2630439

[ref75] Siao W. Wang P. Voigt B. Hussey P. J. Baluska F. (2016). *Arabidopsis* SYT1 maintains stability of cortical endoplasmic reticulum networks and VAP27-1-enriched endoplasmic reticulum–plasma membrane contact sites. J. Exp. Bot. 67, 6161–6171. doi: 10.1093/jxb/erw381, PMID: 27811083 PMC5100027

[ref76] Sjolund R. D. Shih C. Y. (1983). Freeze-fracture analysis of phloem structure in plant tissue cultures: I. the sieve element reticulum. J. Ultrastruct. Res. 82, 111–121. doi: 10.1016/S0022-5320(83)90101-6, PMID: 6848770

[ref77] Strasser R. (2018). Protein quality control in the endoplasmic reticulum of plants. Annu. Rev. Plant Biol. 69, 147–172. doi: 10.1146/annurev-arplant-042817-040331, PMID: 29570364 PMC7039705

[ref79] Thomas E. L. Van der Hoorn R. A. L. (2018). Ten prominent host proteases in plant-pathogen interactions. Int. J. Mol. Sci. 19:639. doi: 10.3390/ijms19020639, PMID: 29495279 PMC5855861

[ref80] Tilney L. G. Harb O. S. Connelly P. S. Robinson C. G. Roy C. R. (2001). How the parasitic bacterium *legionella pneumophila* modifies its phagosome and transforms it into rough ER: implications for conversion of plasma membrane to the ER membrane. J. Cell Sci. 114, 4637–4650. doi: 10.1242/jcs.114.24.4637 11792828

[ref81] Tomkins M. Kliot A. Marée A. F. Hogenhout S. A. (2018). A multi-layered mechanistic modelling approach to understand how effector genes extend beyond phytoplasma to modulate plant hosts, insect vectors and the environment. Curr. Opin. Plant Biol. 44, 39–48. doi: 10.1016/j.pbi.2018.02.002, PMID: 29547737

[ref82] Uchiyama A. Shimada-Beltran H. Levy A. Zheng J. Y. Javia P. A. Lazarowitz S. G. (2014). The *Arabidopsis* synaptotagmin SYTA regulates the cell-to-cell movement of diverse plant viruses. Front. Plant Sci. 5:584. doi: 10.3389/fpls.2014.00584, PMID: 25414709 PMC4222171

[ref002] Ueda H. Tamura K. Hara-Nishimura I. (2015). Functions of plant-specific myosin XI: from intracellular motility to plant postures. Curr. Opin. Plant Biol. 28, 30–38. doi: 10.1016/j.pbi.2015.08.006 26432645

[ref83] Ueda H. Yokota E. Kutsuna N. Shimada T. Tamura K. Shimmen T.. (2010). Myosin-dependent endoplasmic reticulum motility and F-actin organization in plant cells. Proc. Natl. Acad. Sci. U. S. A. 107, 6894–6899. doi: 10.1073/pnas.0911482107, PMID: 20351265 PMC2872430

[ref84] van Bel A. J. E. Furch A. C. U. Will T. Buxa S. V. Musetti R. Hafke J. B. (2014). Spread the news: systemic dissemination and local impact of Ca^2+^ signals along the phloem pathway. J. Exp. Bot. 65, 1761–1787. doi: 10.1093/jxb/ert425, PMID: 24482370

[ref85] van Bel A. J. E. Musetti R. (2019). Sieve element biology provides leads for research on phytoplasma lifestyle in plant hosts. J. Exp. Bot. 70, 3737–3755. doi: 10.1093/jxb/erz172, PMID: 30972422

[ref86] van Bel A. J. E. Schulz A. Patrick J. W. (2022). New mosaic fragments toward reconstructing the elusive phloem system. J. Plant Physiol. 275:153754. doi: 10.1016/j.jplph.2022.153754, PMID: 35753158

[ref87] Verchot J. Pajerowska-Mukhtar K. M. (2021). UPR signaling at the nexus of plant viral, bacterial, and fungal defenses. Curr. Opin. Virol. 47, 9–17. doi: 10.1016/j.coviro.2020.11.001, PMID: 33360330

[ref88] Vitale A. Schnell D. Natasha V. Raikhel R. Maarten J. (2015). Protein Sorting and Vesicle Traffic. Biochemistry and Molecular Biology of Plants, 2nd. New York: John Wiley & Sons, Ltd.

[ref89] Wang P. Hawkins T. J. Richardson C. Cummins I. Deeks M. J. Sparkes I.. (2014). The plant cytoskeleton, NET3C, and VAP27 mediate the link between the plasma membrane and endoplasmic reticulum. Curr. Biol. 24, 1397–1405. doi: 10.1016/j.cub.2014.05.003, PMID: 24909329

[ref90] Yang P. Lüpken T. Habekuss A. Hensel G. Steuernagel B. Kilian B.. (2014). Protein disulfide isomerase like 5-1 is a susceptibility factor to plant viruses. Proc. Natl. Acad. Sci. U. S. A. 111, 2104–2109. doi: 10.1073/pnas.1320362111, PMID: 24481254 PMC3926060

[ref91] Zhang W. Huang L. Zhang C. Staiger C. J. (2021). Arabidopsis myosin XIK interacts with the exocyst complex to facilitate vesicle tethering during exocytosis. Plant Cell 33, 2454–2478. doi: 10.1093/plcell/koab116, PMID: 33871640 PMC8364239

[ref92] Zhang Z. Liu X. Li R. Yuan L. Dai Y. Wang X. (2018). Identification and functional analysis of a protein disulfide isomerase (AtPDI1) in *Arabidopsis thaliana*. Front. Plant Sci. 9:913. doi: 10.3389/fpls.2018.00913, PMID: 30073003 PMC6060501

[ref93] Zhou F.-S. Sun L. Valdés A. E. Engström P. Song Z. T. Lu S. J.. (2015). Membrane-associated transcription factor peptidase, site-2 protease, antagonizes ABA signaling in Arabidopsis. New Phytol. 208, 188–197. doi: 10.1111/nph.13436, PMID: 25919792

