# Supplementary material for: The sieve-element endoplasmic reticulum: A focal point of phytoplasma-host plant interaction?
Source: Front Microbiol. 2023 Feb 2;14:1030414. doi: 10.3389/fmicb.2023.1030414 (PMC9932721; doi:10.3389/fmicb.2023.1030414)
Supplement: Supplementary file 1 [file Image_1.pdf]

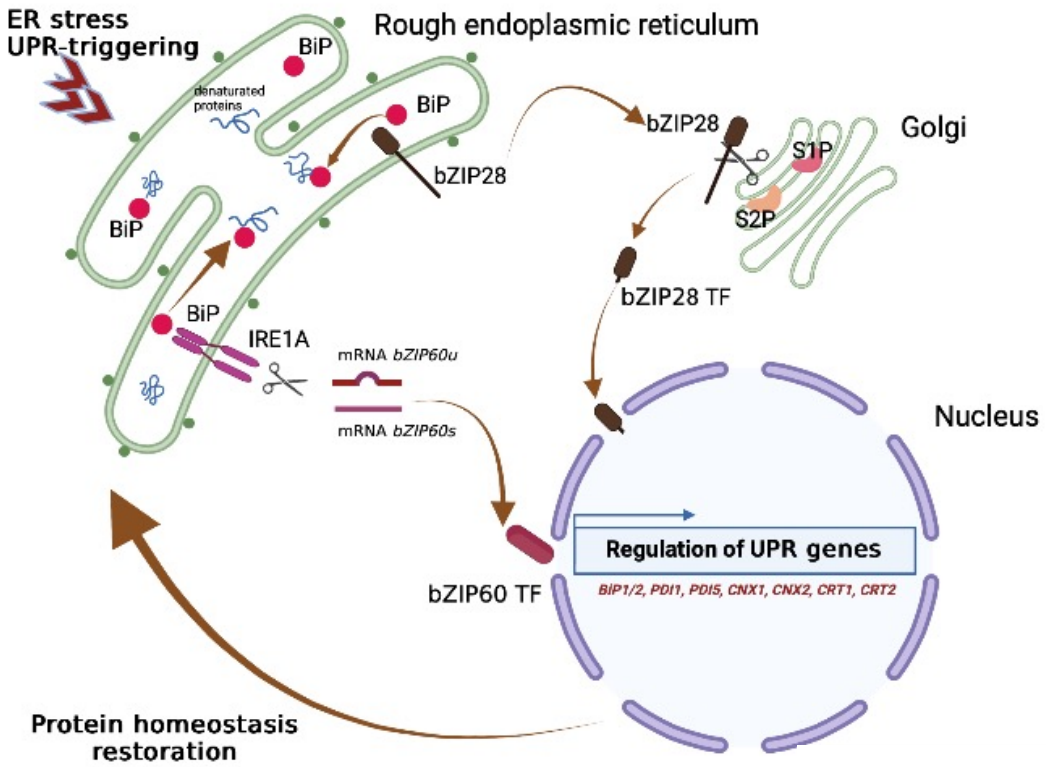


Overview of the unfolded protein response (UPR) in a generic plant cell. The central elements of the UPR signaling network are the signal transducer IRE1A and the activating transcription factor bZIP28, which constitute two branches of the UPR in plant cells. Its specific outputs counteract proteostatic perturbations in the rough endoplasmic reticulum (r-ER). Stress causes overload of misfolded or unfolded proteins in r-ER lumen, provoking the cleavage of BiP protein from the two r-ER-membrane-associated UPR sensors, IRE1A and bZIP28. BiP cleavage leads to the dimerization/oligomerization of IRE1A. IRE1A *trans*-autophosphorylation accomplishes nonconventional messenger RNA (mRNA) splicing and the production of the functional bZIP60 transcription factor (TF) that induces expression of genes involved in r-ER protein homeostasis regulation. Stress also induces bZIP28 translocation to the dictyosomes where it is processed by S1P and S2P proteases to liberate the bZIP28 TF element. Both TFs traffick to the nucleus, where they regulate the expression of the UPR genes. Small scissors indicate IRE1A and bZIP28 processing and the production of the functional TFs. At the transcriptional level, crosstalk exists between the two branches of the UPR signaling pathway, to enhance the r-ER folding capacity.
